# Resident Macrophages Orchestrating Heart Rate

**DOI:** 10.5935/abc.20190041

**Published:** 2019-05

**Authors:** Diego Santos Souza, Tatiane de Oliveira Barreto, Michael Nadson Santos Santana, José Evaldo Rodrigues Menezes-Filho, Jader Santos Cruz, Carla Maria Lins de Vasconcelos

**Affiliations:** 1 Universidade Federal de Sergipe - Fisiologia, São Cristóvão, SE- Brazil; 2 Universidade Federal de Minas Gerais - Bioquímica e Imunologia, Belo Horizonte, MG - Brazil

## Introduction

The electrical conduction system of the heart is essential for maintaining normal heart rhythm and function. This is due to the presence of specialized cells that generate electrical impulses that propagate throughout the heart tissue, quickly and efficiently. This electrical impulse starts at the sinoatrial node (SAN) and propagates sequentially to atrioventricular node (AVN), subsequently being transmitted to the ventricles via specialized conduction pathways. The electrical signals are conducted from cell to cell through a cardiomyocyte permeability control system formed by proteins called connexins, and connexin-43 is the type found in the heart and is associated with the formation of so-called gap junctions. By providing the single electrical connection between the atria and the ventricles, AVN plays an essential role in the dynamics of cardiac contraction. Clinically, when the PR interval is observed in the electrocardiographic recordings, we can correlate the electrical impulse conduction time from its generation in the SAN to the delay in the AVN region, which is called "decremental conduction".^1^ When prolongation of the PR interval or an AV block occurs, which delays excessively or even eliminates the conduction of the electrical impulse from the atria to the ventricles, will result in hemodynamic deterioration, syncope and death, in case the patient is not submitted to the brand heart.^2^

Over the years, several studies have described the macrophages as cells of phagocytic functions that would exclusively act in the immune system protecting the organism against pathogens. However, more recently this paradigm was mainly questioned about the origin of macrophages. Several studies have provided evidence that a subpopulation of macrophages, which originated from embryonic development and do not come from the bloodstream, reside and proliferate in virtually all body tissues and apparently act specifically on each organ. For example, resident macrophages of adipose tissue contribute to the regulation of thermogenesis,^3^ iron recycling in the spleen and liver,^4^ and participate in the process of synaptic maturation in the healthy brain.^5^ Such non-canonical activities emphasize the functional diversity of macrophages and their ability to perform specific tasks in the various tissues, in addition to host defence.^6^ In cardiac tissue, macrophages are intrinsic components of the myocardium in normal functioning, where they appear as spindle cells intercalated between cardiomyocytes, fibroblasts and endothelial cells.^7^

### Macrophages and the heartbeat

Cardiac function depends on the appropriate moment of contraction in several distinct regions, as well as the heart rate.^8^ Hulsmans et al.^9^ observed that mice that had their macrophage fauna weakened, had bradycardia and irregular beats. It is known that connexin-43 is predominant in ventricles of humans and that its reduction promotes bradycardia and AV block,^8^ thus, in observing specialized cells in non-muscular electrical conduction, they found that macrophages are electrically coupled to cardiomyocytes and that these resident macrophages facilitate electrical conduction through the AV node.

**Figure 1:**
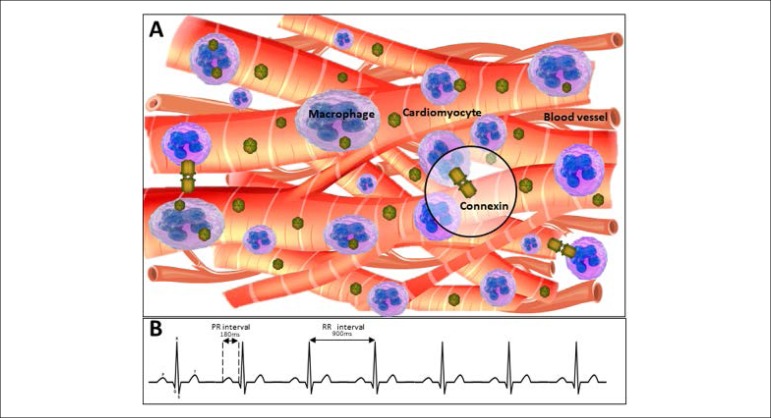
The normal condition of macrophage-cardiomyocyte couplings. Communications between cardiomyocytes and macrophages through connexin-43 (A) promoting normal cardiac rhythm (B).

**Figure 2:**
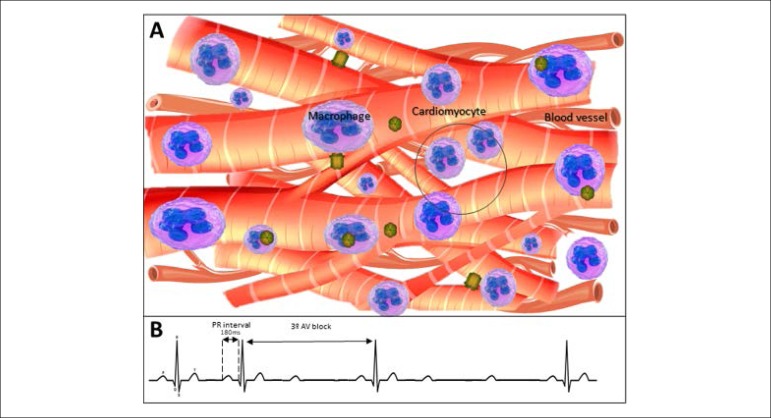
Reduction in the expression of connexins. The coupling between macrophages and cardiomyocytes is decreased due to the reduction of connexin-43 (A) expression promoting electrical conduction pathologies (atrioventricular block of 3º degree - B).

Such conducting cells interleave with macrophages expressing connexin-43 forming additional gap junctions between cardiomyocytes (Figure 1). The investigators observed that the animals that had a reduction of resident macrophages, besides having bradycardia, had AV blockade of 2nd and 3rd degrees (Figure 2),^9^ whose cause in humans is still unknown.^10^ Another intriguing point is that cardiac macrophages have a resting membrane potential of -35 mV on average and depolarize in synchrony with cardiomyocytes. This makes the membrane potential at the rest of the cardiomyocytes more positive and according to the results obtained by computational simulation, accelerate both depolarization and repolarization phases.^9^ The cardioprotective role of cardiac resident macrophages can go beyond the modulation of the electrophysiological properties of the coupled cardiomyocytes. The perivascular localization of cardiac macrophages makes them uniquely positioned to interpret systemic signals in the bloodstream.^10^

### Macrophages and cardiovascular diseases

Monnerat et al.^11^ demonstrated that inflammation caused by type I diabetes causes resident macrophages to secrete interleukin 1β (IL-1β), acting in a paracrine manner, increasing oxidative stress in the surrounding cells and destabilizing the electrical activity of cardiomyocytes provoking lethal ventricular arrhythmias. Moreover, atherosclerotic lesions are currently understood as inducers of important inflammatory processes, which comprise components of the innate and acquired immune systems. Clinical data showed that increased leukocyte count, interleukin-6 (IL-6), tumour necrosis factor (TNF) and IL-1β were at risk of more severe cardiovascular events.

In fact, IL-6 is locally regulated during the coronary occlusion process in patients with acute myocardial infarction with ST-segment elevation.^12-14^

Thus, a possible bias is one in which macrophages contribute to the arrhythmic complications of infectious, atherosclerotic and septicemic diseases, in which their inflammatory responses may interfere with their role in modulating electrical conduction of the cardiomyocyte.^11,12,15^ Research has shown that sepsis is associated with an increased risk of acute and fatal coronary disease, but its cause is still a matter of debate, and acute coronary disease prevention may be an important consideration in post-sepsis medical care.^16,17^

Despite significant advances in prevention and treatment, cardiovascular diseases (CVD) continue to be the most common cause of death in the world. In fact, severe heart failure is more prevalent than cancer.^18^ Several studies have demonstrated that pathological cardiac hypertrophy and fibrosis in heart failure are accompanied by a systemic inflammatory response, infiltration and activation of cells of the immune system.^19^ In view of this, immunotherapies for cardiovascular diseases are on the rise.

The first cardiovascular immunotherapy was developed for the treatment of hypercholesterolemia and its positive results paved the way for the clinical evaluation of anti-inflammatory immunotherapy directed to interleukin 1β. CANTOS (Canakinumab Anti-Inflammatory Thrombosis Outcomes Study) has shown that subcutaneous injections of canakinumab (ACZ885), a human monoclonal antibody that selectively neutralizes IL-1β, significantly reduced levels of systemic inflammatory biomarkers in patients after acute myocardial infarction, reducing risk cardiovascular disease in patients with previous heart attack and inflammatory atherosclerosis.^20^ Another study using CANTOS reinforces this idea and provides strong evidence that the modulation of the IL-6-induced signaling pathway induced by IL-1α is associated with reduced rates of cardiovascular changes and mortality.^13^

It is clear that further studies should be performed to address the actual involvement of resident macrophages in heart diseases. If alterations in macrophages' function are linked to these clinical conditions, immunotherapy with macrophage reprogramming *in situ* could be a reliable form of therapeutic strategy that could be applied to ensure normal cardiac rhythm in patients with signs of arrhythmia.^20,21^ However, what we know so far is that resident macrophages act as "masters", orchestrating the heart rate.

## References

[1] Pastore CA, Pinho JA, Pinho C, Samesima N, Pereira Filho HG, Kruse JCL (2016). III Diretrizes da SociedDE bRsileira de Cardiologia sobre
análise e emissão de laudos
eletrocardiográficos. Arq Bras Cardiol.

[2] Olshansky B (2017). Does First Degree AV Block Have Importance in Patients Considered
for Cardiac Resynchronization Therapy?: Giving It the Third
Degree?. JACC Clin Electrophysiol..

[3] Nguyen KD, Qiu Y, Cui X, Goh YPS, Mwangi J, David T Alternatively activated macrophages produce catecholamines to
sustain adaptive thermogenesis. Nature.

[4] Theurl I, Hilgendorf I, Nairz M, Tymoszuk P, Haschka D, Asshoff M (2016). On-demand erythrocyte disposal and iron recycling requires
transient macrophages in the liver. Nat Med.

[5] Paolicelli RC, Bolasco G, Pagani F, Maggi L, Scianni M, Panzanelli P (2011). Synaptic Pruning by Microglia Is Necessary for Normal Brain
Development. Science.

[6] Davis MJ, Tsang TM, Qiu Y, Dayrit JK, Freij JB, Huffnagle GB Macrophage M1/M2 Polarization Dynamically Adapts to Changes in Cytokine
Microenvironments in Cryptococcus neoformans Infection.

[7] Pinto AR, Paolicelli R, Salimova E, Gospocic J, Slonimsky E, Bilbao-Cortes D (2012). An Abundant Tissue Macrophage Population in the Adult Murine
Heart with a Distinct Alternatively-Activated Macrophage
Profile. PLOS ONE.

[8] Vozzi C, Dupont E, Coppen SR, Yeh HI, Severs NJ (1999). Chamber-related differences in connexin expression in the human
heart. J Mol Cell Cardiol. maio de.

[9] Hulsmans M, Clauss S, Xiao L, Aguirre AD, King KR, Hanley A (2017). Macrophages Facilitate Electrical Conduction in the
Heart. Cell.

[10] Rosenthal N (2017). A guardian of the heartbeat. N Engl J Med.

[11] Monnerat G, Alarcón ML, Vasconcellos LR, Hochman-Mendez C, Brasil G, Bassani RA (2016). Macrophage-dependent IL-1? production induces cardiac arrhythmias
in diabetic mice. Nat Commun.

[12] Held C, White HD, Stewart RAH, Budaj A, Cannon CP, Hochman JS (2017). Inflammatory Biomarkers Interleukin-6 and C-Reactive Protein and
Outcomes in Stable Coronary Heart Disease: Experiences From the STABILITY
(Stabilization of Atherosclerotic Plaque by Initiation of Darapladib
Therapy) Trial. J Am Heart Assoc.

[13] Ridker PM, Libby P, MacFadyen JG, Thuren T, Ballantyne C, Fonseca F (2018). Modulation of the interleukin-6 signalling pathway and incidence
rates of atherosclerotic events and all-cause mortality: analyses from the
Canakinumab Anti-Inflammatory Thrombosis Outcomes Study
(CANTOS). Eur Heart J.

[14] Ridker PM, Lüscher TF (2014). Anti-inflammatory therapies for cardiovascular
disease. Eur Heart J.

[15] Cruz JS, Machado FS, Ropert C, Roman-Campos D (2017). Molecular mechanisms of cardiac electromechanical remodeling
during Chagas disease: Role of TNF and TGF-?. Trends Cardiovasc Med..

[16] Merx MW, Weber C (2007). Sepsis and the heart. Circulation.

[17] Wang HE, Moore JX, Donnelly JP, Levitan EB, Safford MM (2017). Risk of Acute Coronary Heart Disease After Sepsis Hospitalization
in the REasons for Geographic and Racial Differences in Stroke (REGARDS)
Cohort. Clin Infect Dis.

[18] Zannad F (2018). Rising incidence of heart failure demands action. Lancet.

[19] Patel B, Bansal SS, Ismahil MA, Hamid T, Rokosh G, Mack M (2018). CCR2+ Monocyte-Derived Infiltrating Macrophages Are Required for
Adverse Cardiac Remodeling During Pressure Overload. JACC Basic Transl Sci.

[20] Zupancic E, Fayad ZA, Mulder WJM (2017). Cardiovascular Immunotherapy and the Role of
Imaging. Arterioscler Thromb Vasc Biol.

[21] Martini E, Stirparo GG, Kallikourdis M (2018). Immunotherapy for cardiovascular disease. J Leukoc Biol.

